# UNDERSTANDING OLDER ADULTS’ MOTIVATIONS TO ADOPT DIGITAL HEALTH PORTALS

**DOI:** 10.1093/geroni/igad104.3804

**Published:** 2023-12-21

**Authors:** Mimi Trinh, Varitnan Hattakitjamroen, Wendy Rogers

**Affiliations:** University of Illinois--Urbana-Champaign, Champaign, Illinois, United States; University of Illinois--Urbana-Champaign, Champaign, Illinois, United States; University of Illinois--Urbana-Champaign, Champaign, Illinois, United States

## Abstract

Older adults are likely to develop chronic illnesses (e.g., hypertension or high cholesterol), resulting in the need to manage their symptoms. Self-management often involves tracking symptoms, medication taking, nutrition, or exercise. Digital health portals (DHPs) have the potential to fulfill these tracking needs by viewing records of symptoms, doctor’s notes relating to diet or exercise, and reviewing medication regimens. However, DHPs are often not used to their full potential. The goal of this study was to understand what motivates older adults to utilize their DHPs, and what features are needed for health self-management. We conducted a qualitative study consisting of surveys and semi-structured interviews to elicit the thoughts of 26 older participants. Following Self Determination Theory and Unified Theory of Acceptance and Use of Technology Integrated Model, we organized their responses into motivation factors of autonomy, competence, and relatedness as well as facilitators such as perceived usefulness, perceived ease of use, social influence, and facilitating conditions. Using this integrated analysis approach, we identified themes related to health portal experience, motivation, barriers and facilitators, health provider support, and ideal design. These themes provide insights into how the DHPs were used and perceived; why older adults use DHPs; and their overall positive experiences using them. The participants generally found the DHPs to be convenient due to features related to self-management. They also shared their core needs and what could be improved for a better and easier experience. The Integrated Model helps in understanding the foundation needed to motivate the acceptance of DHPs.

